# Supervisors’ transformational leadership style and residents’ job crafting in surgical training: the residents’ views

**DOI:** 10.5116/ijme.622d.e2f6

**Published:** 2022-03-28

**Authors:** Luis Carlos Dominguez, Diana Dolmans, Willem de Grave, Jeroen Donkers, Alvaro Sanabria, Laurents Stassen

**Affiliations:** 1 Department of Surgery, Universidad de la Sabana, Chía, Colombia; 2 Department of Educational Development and Research, School of Health Professions Education (SHE), Faculty of Health, Medicine and Life Sciences (FHML), Maastricht University, Maastricht, the Netherlands; 3 Department of Surgery, Maastricht University Medical Center (MUMC+), Maastricht, the Netherlands

**Keywords:** Surgical training, transformational leadership styles, effectiveness of leadership training, job crafting

## Abstract

**Objectives:**

This study aims to explore the effects of three supervisors’ leadership styles (transformational, transactional, and laissez-faire) on residents’ job crafting.

**Methods:**

Sequential explanatory mixed-methods. First, a purposive sample of residents rated the leadership style of their supervisors and their own job crafting on the Multifactor Leadership Questionnaire and the Dutch Job Crafting Scale. The effects were tested through linear mixed effects regression analysis. Thereafter we conducted semi-structured interviews with residents and conducted a thematic analysis.

**Results:**

A total of 116 residents participated. A transformational style had a positive effect on residents’ job crafting (b = .19, t_(112) _=3.76, p=. 009), whereas the transactional and laissez-faire styles did not. This could be explained by the fact that residents felt a positive influence of the supervisors with such style on the atmosphere for training, on the job resources available to them, and on their modelling function for how to handle the demands of the environment.

**Conclusions:**

A transformational style of the supervisor has a positive effect on residents’ job crafting. Future research should explore the supervisors’ perspective, as well as the effectiveness of leadership training for supervisors with a focus on resident outcomes, such as job crafting.

## Introduction

The problem of residents dropping out of surgical training has received increasing attention because of its negative effects on the individual and on health systems. Residents’ poor well-being in the workplace remains the main reason for the high dropout rate.^1^^-^^4^ Not only supervisors and program coordinators, but also residents are responsible for improving resident well-being. Supervisors play an important role in offering residents support to optimize their learning in the workplace. Residents, in their turn, must undertake actions to optimize and gain control of the workplace for training.^5^^,^^6^ Such actions are in keeping with the concept of job crafting.^7^ Successful job crafting may help more than 80% of residents complete surgical training in different countries.^4^^,^^6^^,^^8^^,^^9^

Originating from the field of proactivity at work, job crafting explains how individuals are able to transform their job actively, instead of simply reacting passively to the working conditions to which they are exposed.^10^ According to the job demands-resources (JD-R) theory, job crafting is exercised when a worker increases two groups of job resources: structural resources (those that foster autonomy) and social resources (those that improve relatedness, e.g. peer collaboration). Workers also craft their jobs by diminishing hindering job demands (e.g., conflicts) and by increasing challenging demands (those that stimulate personal growth and achievement, e.g., problem-solving skills).^11^ When workers craft their jobs, they can become more engaged, which is a positive well-being state as opposed to burnout. Work engagement, ultimately, is related to increased job crafting.^12^

A preliminary study found that when residents’ work engagement was high, their job crafting was negatively correlated with their intention to leave surgical training.^5^ A subsequent study offered insight into how residents craft their jobs to persist in training. They do so through the following six mechanisms: 1) building trust with supervisors, 2) being proactive in the workplace to gain responsibility, 3) seeking help from peers to deal with the demands of training, 4) seeing errors and frustrations as learning opportunities, 5) finding a suitable work-life balance, and 6) searching for challenging surgical tasks.^13^ In the said study, residents acknowledged that supervisors’ leadership played an important role in the six mechanisms. Nonetheless, how supervisors’ leadership drives residents’ job crafting still represents a knowledge gap. There is a need for studies that explore how leaders can influence job crafting in different work contexts. Such studies might help us better understand what actions leaders (i.e., supervisors) could take to support residents in the workplace.

Leaders make use of different styles to lead and motivate people. In the literature, we find three leadership styles that prevail in organizations.^14^ The first is a transformational leadership style (TLS), which is team-oriented and aimed to raise awareness about collective interests in others (e.g., vision of the organization, high standards).^14^ Leaders who have a TLS elicit awareness and knowledge of their own job in others and serve as role models,^14^^-^^16^ they are committed to the organizational culture and justice at work, diminish bullying and burnout and foster work engagement.^14^^,^^15^,^17^^,^^20^Conversely, a transactional leadership style (TrLS) is task-oriented and aims to fulfill objectives, ensure standards and monitor outcomes.^14^ Leaders who have a TrLS offer rewards or punishments to others according to their performance during tasks. They are likely to discourage and decrease empowerment, job satisfaction, and work engagement.^21^^-^^24^Finally, a laissez-faire leadership style (LfLS) is passive. Those who have a LfLS are rarely present and they diminish job satisfaction, productivity, and job effectiveness.^14^^,^^25^

In this study, we explored the effects of three leadership styles in surgical supervisors (transformational, transactional, and laissez-faire) on residents’ job crafting, and the underlying explanations for these results, from the residents’ perspective.

## Methods

We conducted a sequential, explanatory mixed-methods research design.^26^ More specifically, we first measured supervisors’ leadership style and residents’ job crafting using questionnaires to be filled out by residents. Then, we conducted interviews with residents to gain a deeper understanding of why these styles were perceived to affect their proactive behaviors. Such a research approach responds to the need to integrate multiple sources of data that together, by drawing on the strengths of quantitative and qualitative methods, can help explain the complex relationship between leadership and job crafting. ^27^^-^^29^

### Quantitative phase

#### Setting

This study was conducted in Colombia where residents hold full-time positions in healthcare institutions during four years of surgical training. Annual tuition for training in private programs is close to 12,000 USD, which includes 66 hours of duty per week. Three out of 20 residencies in surgery are accredited as high-quality programs in accordance with national standards; two more are in the process of attaining that accreditation. Considering the number of affiliated institutions, each program hosts more than 30 surgical supervisors. Residents’ burnout rate is 33% and almost 11.9% of residents have serious intentions to leave training.^5^

### Participants

We used a purposive sampling procedure. We invited a total of 136 residents from seven surgical programs to participate voluntarily in the quantitative phase. We chose the five programs that were either accredited or in the process of obtaining high-quality accreditation. We included two others that had the longest standing tradition in the country. We explained verbally and in writing to the participants the purposes of the research, as well as the procedures to assure the confidentiality and anonymity of data, at individual and program levels, as well as the further management of information. The Commission for Medical Education of the Universidad de la Sabana granted ethical approval.

### Data collection

We collected data on the supervisors’ leadership style and residents’ job crafting, through validated questionnaires filled out by residents. The main researchers administered paper forms of these questionnaires among residents in their institutions.

### Instruments

#### Supervisors’ leadership styles

We obtained permission to administer the Multifactor Leadership Questionnaire (MLQ-5X) to evaluate the leadership style in the supervisors.^15^ The rater form of the MLQ-5X includes 45 items to assess three styles (transformational, transactional, and laissez-faire).  We asked residents to rate their supervisors in general, hence not their individual supervisors, direct supervisor, or the program director. We chose this approach because, first, residents interacted with multiple supervisors daily; second, the high number of supervisors per program limited the feasibility of each resident to rate them all; and finally, program directors were not fully involved in clinical supervision. Residents were instructed to rate ‘the standard surgeon who supervises residents (in the workplace) in the program that you are enrolled in...’ on a 5-point scale (1=not at all; 5=frequently, if not always). This instruction followed the principle of standard reference by which decision-makers – residents in this case – assign intermediate values to their expectations, ranging from best to worst.^30^ The reliability of the questionnaire (Cronbach's α) is above 0.70.^31^

#### Residents’ job crafting

Residents rated their own job crafting skills on the published version of the Dutch job-crafting scale (DJCS).^7^ The scale includes four sub-scales distributed in 21 items: (1) increase structural job-resources (e.g., autonomy) (5 items); (2) increase social job-resources (e.g., peer collaboration) (5 items); (3) increase challenging job-demands (e.g., those that promote personal growth and personal achievement) (5 items); and (4) decrease hindering demands (e.g., role conflict) (6 items).  Each item is rated on a 5-point scale (1=never; 5=very often). Each of the four subscales had a Cronbach's α >0.70.^7^

#### Statistical analysis

We first calculated the descriptive statistics of the participants. Then, we clustered the individual answers on the MLQ-5X and DJCS to represent the overall scores for each leadership style and job crafting per program, and we calculated their means, standard deviations (SD), and 95% confidence intervals (CI).

We used R (R Core Team, 2019) and lme4^32^ to perform a linear mixed-effects analysis of the relationship between leadership styles and job crafting. As fixed effects, we entered each of the three leadership styles into the model. As a random effect, we entered the program since we wanted to account for differences between programs. We calculated the p values for each fixed effect (leadership styles), and we reported the b estimate, the 95% CI, and the chi-square (df=1) p-value (significance level of <0.05). In general, b estimates around 0.10, 0.25, and 0.40 can be interpreted as a small, medium, and large effects.^33^ For the random effect (program) we reported the variance, standard deviation (SD), the 95% CI, and a simulation-based p-value (significance level of <0.05). We also calculated the interclass correlation (ICC) for the random effect. Finally, we assessed the models’ goodness of fit with the R^2^m (marginal R^2^) and the R^2^c (conditional R^2^) indices.^34^

### Qualitative phase

The qualitative phase took place between January and April 2019. First, we developed an interview guide to explore the quantitative results (Appendix). We discussed and reached a consensus on the main questions in the guide. A qualitative follow-up phase of the quantitative data provided information that allowed us to evaluate the early findings. We recruited 20 residents using a purposive stratified sampling procedure based on demographics (e.g., program and year of training). All interviews were conducted by phone. All participants gave verbal informed consent to be involved in the interviews after we had explained the mechanisms to ensure anonymity, confidentiality, and management of information. The main researcher (LCD) conducted the individual in-depth interviews in Spanish language, using a non-technical language to guide the participants in each leadership style. Upon completion, all interviews were immediately audiotaped and transcribed verbatim. Then, two researchers (LCD and AS), fluent in Spanish language, performed a thematic analysis of all transcripts. With this method, we sought to identify themes within our dataset^35^ and reach thematic saturation. Saturation is reached when no new categories appeared, previous data did not require any more modifications and no additional data were needed.^36^ After ten interviews, both researchers identified the main themes but felt more information was needed to explain some aspects of these themes in depth. We, therefore, conducted four additional interviews, after which LCD and AS felt thematic saturation was reached. All authors subsequently discussed these themes iteratively to reach a consensus.

In this analysis, we acknowledged, through reflexivity, that we as researchers add meaning to the findings. The researchers have different backgrounds, experiences, and perspectives on leadership and residency training, which may have influenced data collection and analysis. Three researchers are surgeons, supervisors, and directors of surgical programs (LCD, LS, AS). Two have extensive experience as educational researchers in workplace-based learning in residency training (DD, WdG) and one author is a statistical expert (JD). Ultimately, our interpretation of the findings was influenced by the concepts of the JD-R theory.^11^ Our different perspectives combined with the said theoretical concepts are thought to benefit the strength of the study and the transferability of its findings. Finally, in all stages of the qualitative phase, we followed recommendations for the translation of information, in our case from Spanish into English language.^37^

## Results

### Quantitative results

We included 116 residents from seven programs which represents 92.6% response rate. Table 1 gives the characteristics of the participants. The mean score of the TLS on the MLQ-5X was 3.39 (0.72). The score of the TrLS was 2.93 (0.46). The score of the LfLS was 2.07 (0.75). The mean scores of the global job crafting on the DJCS was 3.50 (0.41). The scores of the subscale of the DJCS to increase structural resources was 4.35 (0.52); social resources 3.75 (0.66); and challenging demands 3.50 (0.61). Finally, the score of the subscale to diminish hindering demands was 2.59 (0.73).

A linear mixed effects analysis was used to estimate the effect of the supervisors’ leadership styles on residents’ job crafting based on the MLQ-5X and DJCS scores, respectively.

**Table 1:** Descriptive characteristics of programs and participants

Information on participants		N (%) Mean ± SD (Range)
Number of participants (global) Age (mean, standard deviation, range)		116 28.59 ± 2.48 (22-36)
Male (number and percentage) Age (mean, standard deviation, range)		69 (59.48) 28.81 ± 2.77 (22-36)
Female (number and percentage) Age (mean, standard deviation, range)		47 (40.52) 28.27 ± 2 (24-34)
Number and percentage of residents per year of training		N (%)
	Year 1	34 (29.31)
	Year 2	24 (20.69)
	Year 3	32 (27.59)
	Year 4	26 (22.41)
Information on programs		
Number of programs		7
	Average number of residents per training program evaluating their supervisors’ leadership styles (range)	16.6 (10-21)

The results show that there is a positive effect of a TLS on global job crafting (b = .19, t_(112)_ =3.76, p= .009). In general, the difference between programs regarding the effect of leadership style on job crafting was relatively small (ICC ranged between 0.002 and 0.16). We found no significant effects of a TrLS and LfLS on global job crafting. Similarly, a TLS had a positive effect on all job-crafting sub-scales. In three sub-scales (increasing structural and social resources and diminishing hindering demands), the effect was significant (p<0.05). We found no significant effect between a TrLS and any job-crafting sub-scale. A LfLS had only a significant effect on job crafting to decrease hindering demands (p< .0001). Table 2 shows the linear mixed effects analysis of leadership styles and job crafting (globally and per sub-scale). Finally, the fit measures (R^2^m, R^2^c) were relatively low in all models, suggesting that factors other than supervisors’ leadership styles may have influenced residents’ job crafting (Table 2).

### Qualitative results

Six participants were female (42.8%). The distribution of participants by year of training was as follows: year 1 (n=4), year 2 (n=3), year 3 (n=3), and year 4 (n=4). The mean duration of the interviews was 20,35 minutes (5.17). Three predominant themes emerged from the interviews. Table 3 gives an overview of representative quotations.

#### Theme 1: Supervisors’ leadership style influences the atmosphere for training in positive and negative ways

Residents mentioned that supervisors who had a TLS promoted a positive atmosphere for training and showed high standards of patient care. The personal strengths they attributed to such supervisors were altruism, integrity, resilience, and trustworthiness. Residents valued this atmosphere because it made them feel free to discuss their fears and expectations of training, strengthening both their performance (e.g., decision-making and problem-solving skills) and readiness for practice. In residents’ view, these supervisors stimulated them to stay in the program and pursue their training.

Conversely, residents characterized supervisors who had a TrLS as people who actively searched for errors, and were punitive and authoritative, who did not encourage them to take the lead in their own training on the job. In most cases, these supervisors created a hostile atmosphere for training where residents experienced fear and mistreatment, leading to defensive behaviors to hide errors and avoid punishment and to more intentions to leave training.

Finally, residents mentioned that supervisors who had a LfLS showed a lack of commitment to patient care and residents’ education and were perceived to help create a negative atmosphere in the workplace. Moreover, they contributed to more demands for the resident (i.e., more workload), resulting in unsafe care for patients. At the same time, however, the rare presence of these supervisors in the workplace encouraged residents to take more care of patients and to deal with workload and pressure.

#### Theme 2: Supervisors’ leadership style influences the availability of job resources

Residents mentioned that supervisors who had a TLS offered the resident more job resources and challenges in the workplace (e.g., in the form of support, teaching and feedback). Supervisors who had a TrLS and LfLS, by contrast, provided fewer of these resources and challenges, while creating more hindering demands (e.g., workload and pressure). For instance, supervisors who had a TrLS gave poor feedback and instructions and frequently punished residents by limiting opportunities to participate in surgical care and to take on new challenges (such as the opportunity to operate complex patients). In residents’ views, these negative aspects of training led to psychological distress, lack of autonomy and more intentions to leave training.

#### Theme 3: Supervisors’ leadership style serves as a role model for how to handle the demands in the workplace

According to residents, supervisors with a TLS were positive role models. More specifically, these supervisors could handle the work environment and finding solutions to difficult situations effectively, for instance, when surgical complications or conflicts arose at work. Supervisors with a TrLS or an LfLS on the other hand were perceived as negative role models because they created more demands for the resident (in the form of conflicts and ambiguity) while wielding ineffective strategies to solve difficult situations.

**Table 2:** Linear mixed effects analysis of supervisors' leadership styles and residents' job crafting

Job-crafting	Fixed effects						Random effects (adjusted per program)			ICC %	Goodness of fit	
	Transformational style		Transactional style		Laissez-faire style							
	b (95% CI)	*p*	b (95% CI)	*p*	b (95% CI)	*p*	Variance (SD, 95% CI)	Residual (SD, 95% CI)	*p*		R^2^m	R^2^C
Global job crafting	0.19 (0.08 - 0.32)	*0.009*	0.10 (-0.06 - 0.27)	*0.21*	0.07 (-0.01 - 0.17)	*0.10*	0.01 ± 0.11 (0.000 - 0.24)	0.12 ± 0.34 (0.30-0.39)	*0.02*	10.23	0.18	0.26
Increasing structural resources	0.16 (0.04 - 0.29)	*0.009*	0.06 (-0.12 - 0.24)	*0.50*	- 0.10 (-0.20 - 0.005)	*0.06*	0.01 ± 0.11 (0.000 - 0.23)	0.14 ± 0.37 (0.32 -0.43)	*0.04*	8.22	0.15	0.22
Increasing social resources	0.20 (0.005 - 0.40)	*0.04*	0.19 (-0.09 - 0.48)	*0.18*	- 0.05 (-0.22 - 0.11)	*0.51*	0.05 ± 0.22 (0.03 - 0.45)	0.35 ± 0.59 (0.51 - 0.67)	*0.007*	12.88	0.09	0.21
Decreasing hindering demands	0.27 (0.06 - 0.47)	*0.01*	- 0.02 (-0.34 - 0.29)	*0.88*	0.37 (0.19 - 0.56)	*0.0001*	0.001 ± 0.03 (0.000 - 0.23)	0.46 ± 0.68 (0.59 - 0.76)	*0.39*	0.21	0.16	0.16
Increasing challenging demands	0.15 (-0.02 - 0.34)	*0.09*	0.22 (-0.02 - 0.49)	*0.08*	0.06 (-0.08 - 0.21)	*0.39*	0.05 (0.04 - 0.45)	0.29 ± 0.53 (0.46-0.61)	*0.004*	15.79	0.09	0.23

b = b estimate; SD= standard deviation; CI = confidence interval; R^2^m = marginal R squared; R^2^C = conditional R squared

ICC = interclass correlation for the random effect (ICC = variance (program) / (variance (program) + variance (residuals)))

p value: significance level of <0.05

**Table 3:** Quotes from the interviews illustrating the main themes

Supervisors’ leadership style	Theme 1: Supervisors’ leadership style influences the atmosphere for training	Theme 2: Supervisors’ leadership style influences the availability of job resources	Theme 3: Supervisors’ leadership style serves as role model for how to handle the environment
Transformational	These supervisors, undoubtedly, create a better work environment. The hardest part of residency is not the workload, fasting, or the lack of sleep, but having a good relationship with the supervisors and peers. A quiet environment makes it easier for residents to work hard. (Interview #12: 4^th^-year male resident)	This type of leader offers the resident opportunities to be autonomous -within the framework of patient safety- to identify what [the resident] is doing well, and to have confidence ... They [the supervisors] tell him/her: "I want to operate on this patient with you,” or “you are going to operate on this patient." These are ways to strengthen autonomy, which causes the resident to fight his/her own demons [fears]. (Interview #3: 3rd-year male resident)	All of us [residents] have difficult times during residency. If you have a role model, a supervisor with whom to share the anguish, that's good, that can help you to stay in the program. You admire some supervisors, not only because of their surgical capabilities, but also because of their integrity, because they are good human beings ... Sometimes you have big problems, but they can help you to see them smaller. These models make you see the problems from other angles to solve them. (Interview #1: 2^nd^-year female resident)
Transactional	When leadership is based on punishment, the academic and work environments are hostile. Residents’ behavior is based on fear. This is inappropriate practice [professional practice] for the patient. The resident is thinking how to avoid errors, to prevent a negative response from the supervisor. This generates greater stress for the resident ... The resident acts defensively and not proactively in the patient’s favor. (Interview #4: 3^rd^-year female resident)	"These supervisors are focused on the error and do not give feedback to the resident ... so the resident keeps making mistakes. That is not a good way to teach." (Interview #6: 4^th^-year male resident)	I do not want to be like that person [the supervisor]. I do not want that life for me. If you see that your supervisor is rude, bad-tempered, someone who is not able to control his/her anger (such people almost always have personal problems, divorces, and do not spend enough time at home), the resident may say: "this is not the life that I want in the future!" So, the resident leaves the program. (Interview #3: 3^rd^-year male resident)
Laissez-faire	Many supervisors are passive leaders, many of them spend time with residents because it is their obligation. Although they are part of a surgical team, they are not real supervisors, they are not interested [in residents’ education] ... They are indolent and indifferent to the resident ..., so there is no real connection between supervisor and resident ... It is not an enriching environment for learning. (Interview #11: 2^nd^-year female resident)	Residents cannot control the environment well because the supervisor simply leaves the resident alone, does not supervise anything that the resident is doing ... does not teach him/her anything. He/she [the supervisor] does not care if the resident is doing things right or wrong. He/she simply leaves the resident to his own devices. (Interview #13: 4^th^-year female resident)	If you have a disinterested, a passive supervisor who does the minimum required to fulfill, you are getting a bad model. Very little profit is taken from them, because they do not commit, do not make decisions and do not take appropriate care of situations ... They simply expend the least effort possible. (Interview #12: 4^th^-year male resident)

## Discussion

The results of our study indicate that a transformational leadership style (TLS) has a positive effect on residents’ job crafting.  Residents valued supervisors with such a style for their influence on the training atmosphere and on the availability of job resources and because they served as positive role models. Conversely, neither the transactional nor the laissez-faire style was found to have a significant effect on residents’ job crafting. During the interviews, however, residents argued that these supervisors had a negative influence on the training atmosphere, the availability of job resources and role modeling.

We must view these results in relation to the existing research. Our findings echo those of previous research pointing to the positive influence of supervisors with a TLS on the atmosphere in the workplace.^20^^,^^38^^,^^39^ In our study, a safe atmosphere – characterized by a non-punitive and open environment for training – helped create favorable conditions for residents (e.g., trust in the supervisor, less power distance and less fear to discuss expectations). These conditions were felt to be stimulating to search for more opportunities to participate in decision-making, solve complex problems and cope with adversity. In other words, a positive atmosphere is one of the centerpieces of residents’ job crafting. Our results, moreover, suggest that such a positive atmosphere may depend on supervisors’ ability to create a deep connection with residents, which supervisors with a TLS do. These findings tie in nicely with previous studies on the importance of supervisors’ behaviors for a positive learning climate and residents’ well-being.^40^^,^^42^

To our knowledge, this study is the first to explore the impact of a TLS in surgical education, as most studies have hitherto focused on its effects on clinical outcomes (patient safety and team performance).^43^^,^^44^ Moreover, our study emphasizes the importance of a TLS to a crucial aspect of residents’ education, that is, residents’ job crafting, considering the complexity of the surgical work environment for training. Our results also suggest that supervisors must not only offer residents structural resources (e.g., autonomy and responsibility), social resources (e.g., feedback and coaching) and more challenging demands (e.g., participation in complex cases), but they must also encourage them to seek these so that residents can craft their jobs efficiently and improve their performance. These results are in keeping with studies on the effect of a TLS on empowerment and autonomy in healthcare contexts.^22^^,^^45^ We found that supervisors who embrace a TLS can help residents to gain control at work, by demonstrating effective ways to handle hindering demands and stressors (e.g., conflicts, frustrations of training). Few studies have considered judging surgeons who serve as role models by abilities other than their “surgical skills” and “mastery of technique.” The ability to deal with conflicting demands and to cope with adversity are examples of what residents expect to learn from their supervisors beyond the traditional dexterity competences. ^46^^-^^48^ Our findings indicate that supervisors should be aware of their modeling function with respect to these non-technical competences.

Our qualitative findings, on the other hand, suggested that supervisors with a TrLS have a negative influence on the atmosphere for training. Fear and power distance were important factors that explained such a hostile atmosphere. These factors, in turn, serve to illustrate how supervisors who embrace this leadership style are disconnected from residents in the workplace. Ultimately, these supervisors negatively affect the availability of job resources and increase hindering demands (i.e., workload). Other studies have reported similar findings with respect to an authoritative leadership style in the supervisors.^20^^,^^24^^,^^38^^,^^39^^,^^49^ Nonetheless, contrary to what we expected, we identified a positive association between an LfLS and residents’ job crafting to diminish hindering demands. Hypothetically, this could be explained by the fact that residents were forced to take control of patient care and deal with clinical workload in face of poor supervision from the surgeons in charge. These observations deserve further investigation.

We acknowledge that the study has both strengths and limitations. A strength is that it adds information to the available evidence (conducted in non-healthcare settings from non-educational perspectives) supporting the positive relationship between transformational leadership and job crafting.^28^^,^^50^^,^^51^ Moreover, adding a qualitative stage provided more depth and was useful since few studies have focused on the qualitative dimensions of a TLS.^23^^,^^52^ A first limitation is that the data we collected only represented residents’ perspective and, consequently, the study lacks a supervisor perspective. Secondly, we did not study the role of moderators in the relationship between supervisors’ leadership and job crafting. Possible moderators are the organizational culture at the level of departments and institutions, as well as residents’ attributes (e.g., self-efficacy and grit).

This study has implications for practice and research. It is essential that supervisors strive to be transformational leaders, as it will help residents to become skilled job crafters. As suggested by our results, a TLS in the surgical context typifies supervisors who reveal well-developed personal strengths (e.g., integrity and trustworthiness) and commitment to high standards of patient care. They contribute to a positive atmosphere for training in which they offer residents support aimed to strengthen their performance, motivation, and readiness for practice. Finally, they serve as role models for residents, by demonstrating effective behaviors for handling the demands of the work environment. We believe that organizations committed to strengthening a healthy workforce should implement formal training not only in this type of leadership for supervisors, but also in bottom-up strategies for residents to teach them how to optimize job demands and resources (job-crafting training as part of residency training).^12^^,^^20^ Moreover, investing in transformational leadership development for supervisors could help strengthen residents’ job fit in surgical training and reduce burnout and dropout, as has been identified in other work contexts.^39^^,^^53^ Considering the limitations, we call for studies on the organizational influences (e.g., culture and power) on the relationship between supervisors’ leadership and residents’ job crafting. Moreover, supervisors’ perspectives on this topic deserve investigation. Similarly, we invite future studies to explore the effect of transformational leadership training and development for supervisors on residents’ job crafting.

## Conclusions

In conclusion, a TLS of supervisors in surgery has a positive effect on the residents’ capability to optimize their job demands and resources during training to gain control of the work environment. This effect is rooted in the positive influence of that leadership style on the environment for training, on role modeling and on resources for the resident.

### Conflict of Interest

The authors declare that they have no conflict of interest.

## Supplementary material

Supplementary file 1 Appendix. Interview guide for residents
