# Supplementary material for: Supervisors’ transformational leadership style and residents’ job crafting in surgical training: the residents’ views
Source: Int J Med Educ. 2022 Mar 28;13:74–83. doi: 10.5116/ijme.622d.e2f6 (PMC9017501; doi:10.5116/ijme.622d.e2f6)
Supplement: Supplementary file 1 — Appendix. Interview guide for residents [file ijme-13-74-S1.pdf]

Appendix

Interview guide for residents

We surveyed surgical residents from seven programs to assess the relationship between supervisors' leadership style and the ability of residents to control, optimize, and effectively manage their work environment for training. A first finding suggested that an inspirational and stimulating leadership style considerate of residents' needs was positively associated with residents' ability to control, optimize, and effectively manage their work environment.

1. ***Why do you think that this specific type of leadership style by supervisors positively influences residents' abilities to control their own work environment? Please give examples.***

In the same survey we identified a second finding: A leadership style by supervisors oriented to exchanging rewards and punishments with the resident, based on the fulfillment of obligations, objectives, outcomes, and standards at work, was not related to residents' ability to control, optimize and effectively manage their work environment for training.

2. ***Why do you think that this leadership style is not related to residents' ability to control their own work environment? Please give examples.***

Then, we identified that a passive leadership style by supervisors (those who are rarely present and fail to meet residents' expectations and needs) was generally not related to residents' ability to control their work environment. These leadership behaviors, however, did seem to specifically influence in a positive fashion residents' ability to diminish obstacles and hindrances in the workplace.

3. ***Why do you think that this type of leadership style is generally not related to residents' ability to control their own work environment? Please give examples.***
4. ***Why do you think that this type of leadership style is particularly related to residents' ability to diminish obstacles and hindrances at work? Please give examples.***

Finally, do you think these supervisory leadership styles we have discussed influence residents' decision to persist or leave the program? Why?
